# Risk Assessment of Groundwater Contamination: A Multilevel Fuzzy Comprehensive Evaluation Approach Based on DRASTIC Model

**DOI:** 10.1155/2013/610390

**Published:** 2013-12-22

**Authors:** Qiuwen Zhang, Xiaohong Yang, Yan Zhang, Ming Zhong

**Affiliations:** ^1^College of Hydropower and Information Engineering, Huazhong University of Science and Technology, Wuhan 430074, China; ^2^School of Municipal and Geomatics Engineering, Hunan City University, Yiyang 413000, China; ^3^Institute of Disaster Prevention, Beijing 101601, China

## Abstract

Groundwater contamination is a serious threat to water supply. Risk assessment of groundwater contamination is an effective way to protect the safety of groundwater resource. Groundwater is a complex and fuzzy system with many uncertainties, which is impacted by different geological and hydrological factors. In order to deal with the uncertainty in the risk assessment of groundwater contamination, we propose an approach with analysis hierarchy process and fuzzy comprehensive evaluation integrated together. Firstly, the risk factors of groundwater contamination are identified by the sources-pathway-receptor-consequence method, and a corresponding index system of risk assessment based on DRASTIC model is established. Due to the complexity in the process of transitions between the possible pollution risks and the uncertainties of factors, the method of analysis hierarchy process is applied to determine the weights of each factor, and the fuzzy sets theory is adopted to calculate the membership degrees of each factor. Finally, a case study is presented to illustrate and test this methodology. It is concluded that the proposed approach integrates the advantages of both analysis hierarchy process and fuzzy comprehensive evaluation, which provides a more flexible and reliable way to deal with the linguistic uncertainty and mechanism uncertainty in groundwater contamination without losing important information.

## 1. Introduction

As one of the most important types of water resource, groundwater is always impacted by industry, agriculture, mining, and other human activities. Groundwater contamination is a major problem because aquifer and contained groundwater are inherently susceptible to contamination from land use and anthropogenic influence. The risk assessment of groundwater contamination is an effective way to protect groundwater resource.

So far, a great number of achievements of risk assessment of groundwater contamination have been made. Most of them can be categorized into such three classes as overlay and index method, process based method, and statistical method [1–5]. Although there are some other alternative assessment models, many uncertainties are still associated with them. On the one hand, for the lack of knowledge and inability of measurement or calculation, the potential differences between assessment of some factors and their true values often exist. This is one kind of uncertainty resulted from the parameters in risk assessment. On the other hand, groundwater contamination is a complicated process, and the flow and transport in subsurface are relevant to the uncertain natural geological media and hydrographical factors. This is another kind of uncertainty caused by the structures in risk assessment. The most popular technique used to deal with the parametric and structural uncertainties is probabilistic risk analysis; however, the model and description are complex, and it is not easy to express the details. Fault tree is an important way in probabilistic risk analysis, which helps to identify the basic events that lead to system failure. Tartakovsky presented the fault tree for a possible aquifer contamination [2]; it is very useful to know how the contamination occurs. Nevertheless, most of probabilistic risk analyses neglect two types of uncertainty: the epistemic and the aleatory uncertainty. In this case, several other aquifer vulnerability assessment methods, such as GOD rating system, DRASTIC point count system model, SEEPAGE method, AVI rating system, and SINTACS method, have been developed [6–9].

DRASTIC is one of the most widely used models for groundwater vulnerability assessment. It is based on a set of hydrogeological factors that affect the downward migration of pollutants to the aquifer, including depth of the aquifer (*D*), recharge (*R*), aquifer media (*A*), soil media (*S*), topography (*T*), impact of the vadose zone (*I*), and hydraulic conductivity (*C*). The relevance of each hydrogeological factor is indicated by its weight obtained by a committee, which is constant and may not be changed. The final groundwater vulnerability risks are described with some linguistic descriptions as high, moderate, low, and very low. DRASTIC gives a useful index and scale to the model parameters [7]. For better risk assessment in different local issues, several modified models based on DRASTIC are developed. Some modifications are adding of additional parameters, removing of certain parameters, and usage of different ratings and weights for the parameters. The fuzzy rule-based approach is used in groundwater vulnerability assessment and some sensitivity analysis and groundwater vulnerability mapping in spatial context is studied by integrating GIS and neuron-fuzzy technology [10–12]. Thirumalaivasan et al. modified the original DRASTIC model with four parameters such as depth-to-water table, topography, hydraulic conductivity, and impact of vadose zone [13]. Analytic Hierarchy Process (AHP) has been used to compute the ratings and weights of the parameters in AHP-DRASTIC model, in which the construction of pair-wise comparison matrices was decided in consultation with experts and field realities. However, it is difficult for experts to give the score in Satty's scale of importance from 1 to 9 and make a linguistic certainty in vulnerability categories. Visualization DRASTIC is the mental images for human to receive and transmit information based on DRASTIC. Its goal is to output the groundwater visualization mapping and convey meaningful spatial patterns for policymaker [14].

Groundwater environment is a complex fuzzy system with multihierarchy, multicriteria, and multiobject; uncertainty exists both in the parameter and structure. In order to solve this problem of uncertainty in the risk assessment of groundwater contamination, we propose a multilevel fuzzy comprehensive evaluation approach based on the DRASTIC model, which integrates Analysis Hierarchy Process (AHP) and Fuzzy Comprehensive Evaluation (FCE).

This paper is organized as follows. We first apply Sources-Pathway-Receptor-Consequence method to identify the parameters that lead to system failure and then construct the multihierarchy criteria framework of risk assessment of groundwater contamination. Next, the Fuzzy-AHP and Fuzzy-DRASTIC risk assessment model of groundwater contamination is presented. Finally, a case study is applied to illustrate the proposed approach.

## 2. Risk Identification of Groundwater Contamination

Source-pathway-receptor-consequence (S-P-R-C) model is initially presented to explain the linkage between hazard and risk of flood. In risk management, the term “hazard” means an event that could cause harm, and the term “risk” is used to simply express the probability of something happening. Hazard is a physical event, phenomenon, or human activity with the potential to result in harm; it does not automatically lead to a harmful outcome, but identification of a hazard does mean that there is a possibility of harm occurrence.

S-P-R-C is a simple conceptual model used to represent the system or process that leads to a particular consequence. In these components, source, pathway, and receptor refer to the physical process, while consequence is a matter of societal values.

In the study of groundwater vulnerability, when contaminants, as the origin of a hazard, are released into the environment, they typically migrate through the upper unsaturated soil to reach and pollute groundwater. In terms of groundwater contamination risk assessment, a description of the nature of the hazard will be needed to assess the potential consequences of a groundwater contamination occurrence.

Groundwater contamination risk can be dependent on the interaction of source variables in DRASTIC model; for example, depth of the aquifer, recharge, aquifer media, soil media, topography, impact of the vadose zone, and hydraulic conductivity may all possibly influence the contamination of groundwater. In these circumstances, the derivation of the probability of groundwater contamination can be complex, which results in analysis of the source and pathway variables described as probability distributions with associated dependencies. Consequences refer to the impact from a risk. In groundwater vulnerability, consequences are described as human health and other environmental pollution.

The source-pathway-receptor-consequences model is a convenient tool to consider how the hazard leads to the risk and how the risk occurs. According to this framework, the risks of groundwater contamination are identified as shown in Table 1.

## 3. Risk Assessment Methodology of Groundwater Contamination Based on AHP and Fuzzy Theory

Risk assessment is often complex and multifactor and involves many different stakeholders with different priorities or objectives. Most people, when confronted with such problems, will attempt to use intuitive or heuristic approaches to simplify the complexity until the problem seems more manageable. In this process, important information may be lost, opposing viewpoints may be discarded, and elements of uncertainty may be ignored. Current risk analyses typically offer little guidance on how to integrate or judge the relative importance of information from each source. In addition, information comes in different forms such as quantitative estimation and qualitative judgment. A systematic methodology to combine both quantitative and qualitative input of risk factors therefore should be developed.

Multilevel fuzzy comprehensive evaluation is an advanced method, in which both fuzzy analytic hierarchy process and fuzzy comprehensive evaluation are integrated together. Based on the index system of risk evaluation, the weights of all risk factors are improved by applying the fuzzy analytic hierarchy process, and then by using the multilevel fuzzy comprehensive evaluation method, the comprehensive value of the risk is calculated.

In DRASTIC model, seven risk indexes are considered in one layer. In fact, groundwater contamination is a comprehensive process that results from geological, hydrological, and environmental factors, and each of them plays different roles and different importance in this process. Constructing a multilayer model based on the risk identification is the first step in groundwater contamination assessment.

### 3.1. Determining Weights by AHP

AHP is a systematized and hierarchical technique of qualitative and quantitative analysis used to deal with complex decisions. Based on mathematics and psychology, it was first developed by Satty in 1970s and has been extensively studied and refined since then [15–19]. The basic idea of AHP is to determine the relative importance through pairwise comparison matrix after constructing a hierarchy expressed by quantification. The proposed AHP procedure of risk assessment in groundwater contamination is defined as follows.

(*1) Construction of Risk Index System*. According to the achievements of groundwater contamination in previous researches, this paper establishes the index system of risk assessment of groundwater contamination based on DRASTIC model that includes seven factors such as depth of the aquifer (*D*), recharge (*R*), aquifer media (*A*), soil media (*S*), topography (*T*), impact of the vadose zone (*I*), and hydraulic conductivity (*C*). Let *U* be the set of risk assessment index as follows:

(1) $$\begin{array}{c} U=\left\{D,R,A,S,T,I,C\right\}. \end{array}$$

According to the contamination resources, these seven factors are divided into two main types: permeation factors (*U*_1_) and conduction factors (*U*_2_) as follows:

(2) $$\begin{array}{c} U=\left\{U_{1},U_{2}\right\},\\ U_{1}=\left\{A,S,I\right\},\\ U_{2}=\left\{D,R,T,C\right\}. \end{array}$$

(*2) Definition of Remark Set*. Remark set is *V* = (*V*_1_ *V*_2_ ⋯ *V*_*n*_), and *V*_*t*_ (*t* = 1,2,…*n*) shows the remark from low to high level of risk. In this paper, the remark set is defined as 5 grades as shown in formula (3) and Table 2

(3) $$\begin{array}{c} V=\left\{V_{1},V_{2},V_{3},V_{4},V_{5}\right\}. \end{array}$$

(*3) Establishment of Reciprocal Judgment Matrix and Weight Sets*. The reciprocal judgment matrix can be described as follows:

(4) AM×M=A1  A2  ⋯  AMA1A2⋮AM[1a12⋯a1Ma211⋯a2M⋮⋮⋯⋮aM1aM2⋯1],

where *a*_*ij*_ (*i* = 1, 2, … , *m*, *j* = 1, 2, … , *m*) is the pairwise relationship between factor *a*_*i*_ at the *i*th row and factor *a*_*j*_ at the *j*th column in the same layer, which can be used to indicate the relative importance of *a*_*i*_ and *a*_*j*_ after their comparison [15, 20].

The work by Aller et al. shows the assigned weights for DRASTIC features. Based on these weights, the reciprocal pairwise relationship is established [7]. However, it is not easy for experts to give the score in Satty's scale of importance from 1 to 9; two modified scales are introduced in this paper.

*(A) 0–2 Scale*. In many practical cases, the experts' preferences are uncertain and they are reluctant or unable to make numerical comparisons. In this paper, the first step is to identify the scale according to 0–2 method and then convert it into 1–9 scale by formulation.

Based on the weights assigned by Aller et al. [7], the pairwise comparison matrixes of the permeation factors (*U*_1_) and conduction factors (*U*_2_) in DRASTIC model by 0–2 scale are produced; the results are shown in Tables 3 and 4, respectively.

After establishment of the above reciprocal matrixes, the weights of every factor in DRASTIC model can be determined by solving the characteristic vectors of these matrixes. Firstly, the weight vectors have to be normalized into the range of [0, 1] by formula (5),

(5) $$\begin{array}{c} w=\frac{w_{i}}{\sum_{1}^{n}w_{i}}. \end{array}$$

Suppose *w* = (*w*_1_, *w*_2_, *w*_3_, …, *w*_*i*_) are weights of all factors, where 0 < *w*_*i*_ ≤ 1, ∑_1_^*n*^ *w*_*i*_ = 1, *A*_*m*×*m*_•*W* = *λ*_max⁡_•*W*. The weight vectors of sublevel factor are shown as follows:

(6) $$\begin{array}{c} W_{1}=\left[0.2583,0.1047,0.6370\right],\;\lambda_{1}=3.0385,\\ W_{2}=\left[0.5650,0.2622,0.0553,0.1175\right],\;\lambda_{2}=4.1170. \end{array}$$

Supposing that all the seven factors in DRASTIC model are equal, the weight vector of the top layer is as follows:

(7) $$\begin{array}{c} W=\left(W_{1},W_{2}\right)=\left(\frac{3}{7},\frac{4}{7}\right)=\left(0.43,0.57\right). \end{array}$$

*(B) 0.1–0.9 Scale*. In comparison between two factors, it is difficult to give a scale number exactly. Fuzzy numbers depict the physical world more realistically than single valued numbers. With 0.1–0.9 scales as shown in Table 5, triangular fuzzy numbers are applied to construct pairwise comparison matrix of factors in DRASTIC model. The results are shown in Tables 6 and 7, respectively.

Similar as the method of 0–2 scale, after establishment of the above reciprocal matrixes in Tables 6 and 7, the weights of every factor in DRASTIC model can also be determined by solving the characteristic vectors of these matrixes. The weight vectors based on 0.1–0.9 scale are shown as follows:

(8) $$\begin{array}{c} W_{1}=\left[0.3415,0.2801,0.3784\right],\;\lambda_{1}=1.5319,\\ W_{2}=\left[0.3239,0.2907,0.1388,0.2466\right],\;\lambda_{2}=1.8672. \end{array}$$

Supposing that all the seven factors in DRASTIC model are equal, the weight vector of the top layer is as follows:

(9) $$\begin{array}{c} W=\left(W_{1},W_{2}\right)=\left(\frac{3}{7},\frac{4}{7}\right)=\left(0.43,0.57\right). \end{array}$$

(*4) Consistency Ratio*. In order to control the result of the method, the consistency ratio for the hierarchy should be calculated. The deviations from consistency, which are called C.I, are expressed by

(10) $$\begin{array}{c} \text{C}.\text{I}=\frac{\lambda_{\max}-n}{n-1}, \end{array}$$

where *λ*_max⁡_ is the principal eigenvalue of the judgment matrix and *n* is the order of the judgment matrix.

The consistency ratio (C.I) is used to directly estimate the consistency of pairwise comparisons matrix. The closer inconsistency index tends to zero, the greater the consistency is.

### 3.2. Determining Membership Degree by Fuzzy Theory

**Figure 1:**
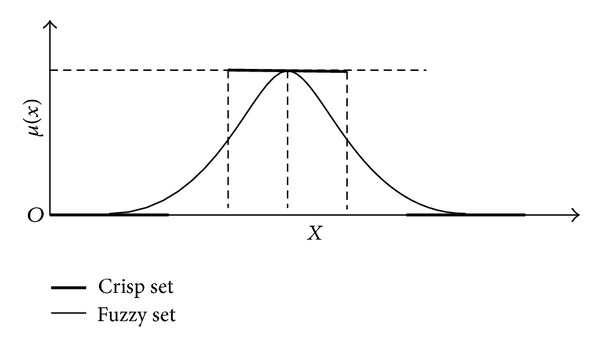
Crisp sets and fuzzy sets.

Fuzzy theory has been successfully applied in a variety of fields with uncertainty such as control of complex systems and expert systems [21–24]. For any set *X*, a membership function on *X* is any function from *X* to the interval [0, 1]. For an element “*x*” of *X*, the membership represents the degree of membership of “*x*” in “*A*.” When the membership *A*(*x*) is near “1,” it is said that there is a high possibility that “*x*” belongs to “*A*”; on the other hand, when the membership *A*(*x*) is close to “0,” there is a low possibility that “*x*” belongs to “*A*.” The difference between membership of fuzzy sets and crisp sets is shown in Figure 1.

**Figure 2:**
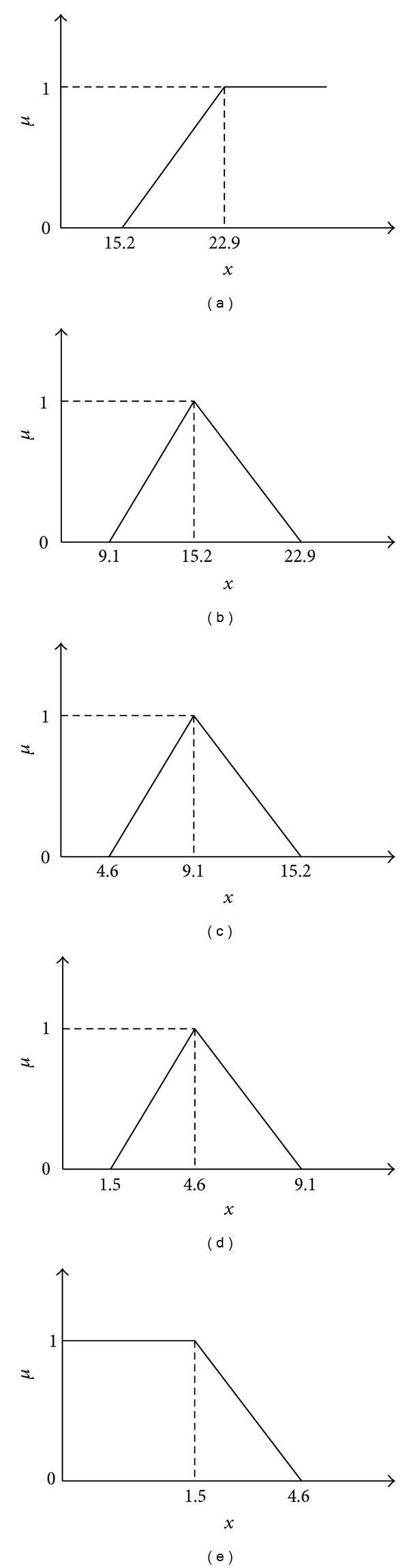
Triangular membership functions of depth of the aquifer (*D*).

There are many uncertainties in groundwater contamination. It is very suitable to use fuzzy theory to assess its risk. On the one hand, the membership degree can be used to describe the risk level in Table 2. For example, the membership degree of the first level with regard to the fuzzy concept of “most difficult to be polluted” (*V*_1_) is assumed to be 0, and the membership degree of the fifth level with regard to the fuzzy concept of “easy to be polluted” (*V*_5_) is assumed to be 1. On the other hand, the membership degrees of each factor in DRASTIC model can be used to express their relationships with influences so that the final fuzzy nexus matrix can be constructed. Because there are two types of factor such as continuous variable and discrete variable, in this paper, we adopt different membership functions to obtain them, respectively.

(*1) Continuous Variable*. The probability distribution function *F*(*x*) of continuous variable *x* in interval [*a*, *b*] is calculated as follows:

(11) $$\begin{array}{c} F\left(x\right)=\overset{x}{\underset{a}{\int }}f\left(x\right)dt,\;x\in \left[a,b\right], \end{array}$$

where *f*(*x*) is the membership function. In this paper, triangular membership function, which is one of the most widely used linear models, is adopted to express the membership function of continuous factors in DRASTIC model such as *D*, *R*, *T*, and *C*. In order to illustrate the process, we choose the depth of the aquifer (*D*) in DRASTIC model to explain. Firstly, we confirm the boundary value and the medium value of the factor in five remark sets. As shown in Figure 2(a), when *D* is larger than or equal to 22.9 m, there is the highest possibility that *D* belongs to remark set *V*_1_, and the membership degree has the value of  “1.” When *D* is less than 15.2 m, there is the lowest possibility that *D* belongs to remark set *V*_1_ and the membership degree has the value of “0.” *μ*_1_ is the membership degree that *D* belongs to remark set *V*_1_. In this way, the membership degree that *D* belongs to every set of the five remarks can be calculated.

The membership functions of depth of the aquifer (*D*) in DRASTIC model are shown as follows:

(12) $$\begin{array}{c} \mu_{1}=\{\begin{array}{cc} 0, & x<15.2\\ \frac{10x-152}{77}, & 15.2\leq x<22.9\\ 1, & x\geq 22.9, \end{array}\\ \mu_{2}=\{\begin{array}{cc} 0, & x<9.1\\ \frac{10x-91}{61}, & 9.1\leq x<15.2\\ \frac{229-10x}{77}, & 15.2\leq x<22.9\\ 0, & x\geq 22.9, \end{array}\\ \mu_{3}=\{\begin{array}{cc} 0, & x<4.6\\ \frac{10x-46}{45}, & 4.6\leq x<9.1\\ \frac{-10x+152}{61}, & 9.1\leq x<15.2\\ 0, & x\geq 15.2, \end{array}\\ \mu_{4}=\{\begin{array}{cc} 0, & x<1.5\\ \frac{10x-15}{31}, & 1.5\leq x<4.6\\ \frac{91-10x}{45}, & 4.6\leq x<9.1\\ 0, & x\geq 9.1, \end{array}\\ \mu_{5}=\{\begin{array}{cc} 1, & 0\leq x<1.5\\ \frac{46-10x}{31}, & 1.5\leq x<4.6\\ 0, & x\geq 4.6. \end{array} \end{array}$$

For an example, supposing the burial depth of groundwater (*D*) is 10 m, according to the continuous membership functions in (12), the membership degrees are calculated as follows:

(13) $$\begin{array}{c} \mu_{1}=0,\\ \mu_{2}=\frac{10x-91}{61}=0.15,\\ \mu_{3}=\frac{-10x+152}{61}=0.85,\\ \mu_{4}=0,\\ \mu_{5}=0. \end{array}$$

So, the membership vector of *D* = 10 m to the five risk remark categories can be described by (0, 0.15, 0.85, 0, 0).

(*2) Discrete Variable*. The possible values of discrete variable *x* are some special values in the interval. Supposing *X* is the set of discrete variable *x* and *x*_1_, *x*_2_,…, *x*_*n*_ are the possible values of *x* in [*a*, *b*], where *x*, *x*_1_, *x*_2_,…, *x*_*n*_ ∈ *X*, the probability distribution function *F*(*x*) of *x* in interval [*a*, *b*] is calculated as follows:

(14) $$\begin{array}{c} F\left(x\right)=P\left(x_{1}\right)+P\left(x_{2}\right)+\cdots +P\left(x_{n}\right), \end{array}$$

where *P*(*x*_*n*_) is the probability of the discrete value *x*_*n*_. According to the probability distribution of discrete variables, various states of the discrete factors in DRASTIC model such as *A*, *S*, and *I* can be expressed by vector format. For an example, the membership function of soil media (*S*) is shown in Table 8.

For an example, supposing the soil media (*S*) consists of 30% sand, 30% loam, and 40% clay loam, based on the discrete function in Table 8, the membership vector of *S* to the five risk remark categories is calculated as follows:

(15) (0,0,0,0,0)+(0,0,0,0,0)+(0,0,0,0,1×30%)  +(0,0,0,0,0)+(0,0,0,0,0)+(0,0,0,0,0)  +(0,0,1×30%,0,0)+(0,0,0,0,0)  +(0,1×40%,0,0,0)+(0,0,0,0,0)+(0,0,0,0,0) =(0,0.4,0.3,0,0.3).

### 3.3. Fuzzy Comprehensive Evaluation

According to the fuzzy comprehensive evaluation theory, the risk of groundwater contamination can be assessed from bottom to top layer by layer, and the evaluation index of the *k*th layer is membership degree of the *k* − 1 layer. Therefore, fuzzy comprehensive evaluation can be got as follows:

(16) $$\begin{array}{c} S=W•R, \end{array}$$

where “•” is a compound operator in fuzzy matrix. There are four models in practice, such as *M*(∧, ∨), *M*(•, ∨), *M*(•, ⊕), and *M*(∧, ⊕). According to the evaluation factors, we select *M*(•, ⊕) as the comprehensive evaluation function.

There are many methods to determine the risk level from the assessment result vector. Two most popular ones of them are maximum membership degree principle (Principle 1) and quantification principle (Principle 2).

(1) *Maximum membership degree principle*. This is a simple and widely used principle on the membership degree matrix. The elements in the vector of evaluation result stand for the membership degree to risk level. According to maximum membership degree principle, the risk element that is corresponding to the maximum number in the evaluation result vector is considered as the final assessment result.
(2) *Quantification principle*. The remark set is a fuzzy set; when the maximum membership degree is close to the second maximum one, it is not proper to make the final decision by maximum membership degree principle. In order to get a more accurate and specific result, the quantification principle arises to deal with the remark set. Suppose that *C*_*h*_ is the high limit set of intervals, *C*_*l*_ is the low limit set of intervals, and *C*_*m*_ is the middle level set of intervals,
  (17) $$\begin{array}{c} C_{m}=\frac{C_{l}+C_{h}}{2}\in \left(C_{l},C_{h}\right). \end{array}$$

In risk assessment of groundwater contamination, if the contaminant in question is carcinogenic, its impacts on human health can be quantified by the Excess Lifetime Cancer Risk (ELCR) factor. According to the U.S. Environmental Protection Agency, the levels of a carcinogen in groundwater are considered safe if ELCR is within the range of [0.0001, 0.000001]. According to the quantification principle, supposing *V*_1_ ∈ [10^−6^, 5 × 10^−5^], *V*_2_ ∈ [5 × 10^−5^, 10^−4^], *V*_3_ ∈ [10^−4^, 1.5 × 10^−4^], *V*_4_ ∈ [1.5 × 10^−4^, 2 × 10^−4^], *V*_5_ ∈ [2 × 10^−4^, 2.5 × 10^−4^], in this case, *V*_1_ and *V*_2_ are considered to be the safe grade, while *V*_3_, *V*_4_, and *V*_5_ are considered to be the dangerous grade [7]

(18) Cl=(10−6,5×10−5,10−4,1.5×10−4,2×10−4),Cm=(2.5×10−5,7.5×10−5,1.25×10−4, 1.75×10−4,2.25×10−4),Ch=(5×10−5,10−4,1.5×10−4,2×10−4,2.5×10−4).

Accordingly, after the quantification, we get three typical results, in which *V*_*l*_, *V*_*m*_, *V*_*h*_ ∈ (*C*_*il*_, *C*_*ih*_),

(19) $$\begin{array}{c} S_{h}=\frac{\sum_{i=1}^{n}b_{i}c_{hi}}{\sum_{i=1}^{n}b_{i}},\\ S_{l}=\frac{\sum_{i=1}^{n}b_{i}c_{li}}{\sum_{i=1}^{n}b_{i}},\\ S_{m}=\frac{\sum_{i=1}^{n}b_{i}c_{mi}}{\sum_{i=1}^{n}b_{i}}. \end{array}$$

## 4. Case Study

This section illustrates an application of the above methodology with AHP and fuzzy theory integrated to assess the risk of the five samples from Dalian peninsula region in the northeast of China. The data come from the case study of F-DRASTIC method in reference [25]. All the seven factors in DRASTIC model of the five samples are listed in Table 9.

As an example, we consider sample 1 in detail. According to the above method of membership degree determination for continuous and discrete variables, the membership degree of sample 1 is shown in Table 10. Integrated with the weights of factors in DRASTIC model, their normalized fuzzy nexus matrixes are established as shown in Table 11.

Then, by fuzzy mapping as follows, the first level of fuzzy comprehensive evaluation is carried out,

(20) $$\begin{array}{c} W_{1}=\left(0.2583,0.1047,0.637\right),\\ W_{2}=\left(0.565,0.2622,0.0553,0.1175\right),\\ S_{1}=W_{1}•R_{1}=\left(0,0,0.5423,0.2884,0.1693\right),\\ S_{2}=W_{2}•R_{2}=\left(0.6707,0.0118,0.1573,0.1187,0.0415\right). \end{array}$$

Considering *S*_*i*_ (*i* = 1, 2) as single fuzzy judgment matrix, the second level fuzzy comprehensive evaluation is shown as follows:

(21) $$\begin{array}{c} S=W•\left[\begin{array}{c} S_{1}\\ S_{2} \end{array}\right]=\left(0.3823,0.0067,0.3229,0.1917,0.0964\right). \end{array}$$

Finally, according to the maximum membership degree principle, 0.3823 is the maximum membership degree that is corresponding to the risk level of *V*_1_, so the risk assessment result of sample 1 is “most difficult to be polluted (*V*_1_).”

According to the quantification principle, the *S*_*h*_, *S*_*m*_, and *S*_*l*_ are calculated as follows:

(22) $$\begin{array}{c} S_{h}=1.0897\times 10^{-4}\in V_{3},\\ S_{m}=1.0566\times 10^{-4}\in V_{3},\\ S_{l}=8.0660\times 10^{-5}\in V_{2}. \end{array}$$

These results show that the risk of groundwater contamination in sample 1 is probably between the level of *V*_2_ and *V*_3_. In this paper, we take *S*_*h*_ as the final risk assessment result of quantification principle, which means that this sample is “difficult to be polluted (*V*_3_).”

The risks of other samples are shown in Table 12. The risk order of these samples from low to high is 1, 5, 4, 2, and 3. The results show that sample 1 is the most difficult to be polluted by groundwater and has the best conditions of geology and hydrology.

## 5. Conclusions

Risk assessment of groundwater contamination is one of the most important tasks in the feasibility study of water supply. According to the concept of risk, this study starts by applying resources-pathway-receptor-consequence method in groundwater contamination to identify the risk factors. Based on DRASTIC model, we proposed a multilevel fuzzy comprehensive evaluation approach for the risk assessment of groundwater contamination, in which the weights and membership degrees of the risk factors are established with fuzzy-AHP and fuzzy-DRASTIC integrated together. This framework can be used to make decision under uncertainties of parameters and complexity of possible transitions in groundwater contamination.

This study provides two types of scale for decision maker to describe the pairwise comparison matrixes, such as 0–2 scale and 0.1–0.9 scale. It is much easier for experts to give the score than traditional Satty's scale. The results of risk assessed by the approach proposed in this paper are expressed in a vector, whose elements stand for the membership degrees to the five levels of groundwater contamination risk. There are also two choices for managers to make the final decision of risk assessment in this study: maximum membership degree principle and quantification principle.

The proposed approach integrates AHP and fuzzy comprehensive evaluation together; it provides a more flexible and reliable way to deal with both the linguistic uncertainty and mechanism uncertainty in groundwater contamination. In addition to the application in risk assessment of groundwater contamination, this approach also can be used to solve the problems of complex and multifactor process without losing any important information.

## Figures and Tables

**Table 1:** S-P-R-C model of groundwater contamination.

Source	Pathway	Receptor	Consequences
*D* *R* *A* *S * *T * *I * *C *	Path 1 Path 2 Path 3	People Property Environment	Human health Environmental pollution

Path 1: the contamination migrates out of the region.

Path 2: the contamination bypasses the reactive barrier and enters the protected zone.

Path 3: the contamination migrates to the region intercepted by the permeable reactive barrier.

**Table 2:** Relationship between linguistic variables and risk evaluation levels.

Risk level	Linguistic variable
*V*_1_	Most difficult to be polluted
*V*_2_	Very difficult to be polluted
*V*_3_	Difficult to be polluted
*V*_4_	Slightly easy to be polluted
*V*_5_	Easy to be polluted

**Table 3:** Pairwise comparison matrix of *U*_1_ based on 0–2 scale.

Factor	*A *	*S *	*I *	Order index
*A *	1	2	0	3
*S *	0	1	0	1
*I *	2	2	1	5

**Table 4:** Pairwise comparison matrix of *U*_2_ based on 0–2 scale.

Factor	*D *	*R *	*T *	*C *	Order index
*D *	1	2	2	2	7
*R *	0	1	2	2	5
*T *	0	0	1	0	1
*C *	0	0	2	1	3

**Table 5:** Definition of 0.1–0.9 scale.

Scale	Definition
0.1	B is extremely more important than A
0.2	B is strongly more important than A
0.3	B is more important than A
0.4	B is a little more important than A
0.5	A is as important as B
0.6	A is a little more important than B
0.7	A is more important than B
0.8	A is strongly more important than B
0.9	A is extremely more important than B

**Table 6:** Pairwise comparison matrix of *U*_1_ based on 0.1–0.9 scale.

Factor	*A *	*S *	*I *	Order index
*A *	0.5000	0.6100	0.4798	1.5898
*S *	0.4482	0.5000	0.3593	1.3075
*I *	0.6719	0.5750	0.5000	1.7469

**Table 7:** Pairwise comparison matrix of *U*_2_ based on 0.1–0.9 scale.

Factor	*D *	*R *	*T *	*C *	Order index
*D *	0.5000	0.5515	0.8935	0.6167	2.5617
*R *	0.4081	0.5000	0.7986	0.6025	2.3092
*T *	0.1101	0.2449	0.5000	0.3248	1.1798
*C *	0.4098	0.3538	0.6968	0.5000	1.9604

**Table 8:** Membership function of soil media (*S*).

No.	Membership degree
1	(0, 0, 0, 0, 1)
2	(0, 0, 0, 0, 1)
3	(0, 0, 0, 0, 1)
4	(0, 0, 0, 0, 1)
5	(0, 0, 0, 1, 0)
6	(0, 0, 0, 1, 0)
7	(0, 0, 1, 0, 0)
8	(0, 1, 0, 0, 0)
9	(0, 1, 0, 0, 0)
10	(1, 0, 0, 0, 0)
11	(1, 0, 0, 0, 0)

**Table 9:** Factor values of the samples in the case study.

Sample no.	*D* (m)	*R* (mm)	*A *	*S *	*T* (%)	*I *	*C* (m/day)
1	61	170.2	5	2	3	9	4.92
2	12	64.3	7	3	6	9	1.64
3	8	45.7	3	10	4	5	24.6
4	16	85.1	3	6	2	7	13.2
5	55	128.3	6	1	7	5	0.41

**Table 10:** Membership degrees of the factors in sample 1.

Risk factor	Membership degree
*D* = 61 m	(1, 0, 0, 0, 0)
*R* = 170.2	(0, 0, 0.60, 0.40, 0)
*A* = 5	(0, 0, 0.25, 0.50, 0.25)
*S* = 2	(0, 0, 0, 0, 1)
*T* = 3	(0, 0, 0, 0.25, 0.75)
*I* = 9	(0, 0, 0.75, 0.25, 0)
*C* = 4.92 m/d	(0.90, 0.10, 0, 0, 0)

**Table 11:** Priority weights in AHP system and membership degrees in fuzzy system.

Risk level	Main factors	Weight	Subfactors	Weight	Fuzzy relationship				
					*V*_1_	*V*_2_	*V*_3_	*V*_4_	*V*_5_
*V*_1_ *V*_2_ *V*_3_ *V*_4_ *V*_5_	Permeation	0.43	*A *	0.2583	0.00	0.00	0.25	0.50	0.25
			*S *	0.1047	0.00	0.00	0.00	0.00	1.00
			*I *	0.6370	0.00	0.00	0.75	0.25	0.00
	Conduction	0.57	*D *	0.5650	1.00	0.00	0.00	0.00	0.00
			*R *	0.2622	0.00	0.00	0.60	0.40	0.00
			*T *	0.0553	0.00	0.00	0.00	0.25	0.75
			*C *	0.1175	0.90	0.10	0.00	0.00	0.00

**Table 12:** Risk results of multilevel fuzzy comprehensive evaluation of the samples in case study.

Sample no.	Risk factors							Evaluation results					Final results	
	*D *	*R *	*A *	*S *	*T *	*I *	*C *	*V*_1_	*V*_2_	*V*_3_	*V*_4_	*V*_5_	Principle 1	Principle 2
1	61	170.2	5	2	3	9	4.92	**0.3823**	0.0067	0.3229	0.1917	0.0964	*V*_1_	1.0897 × 10^−4^
2	12	64.3	7	3	6	9	1.64	0.1019	0.2530	**0.4334**	0.1444	0.0672	*V*_3_	1.4109 × 10^−4^
3	8	45.7	3	10	4	5	24.6	0.0912	0.1319	**0.4797**	0.2364	0.0608	*V*_3_	1.5218 × 10^−4^
4	16	85.1	3	6	2	7	13.2	0.0354	**0.5305**	0.2206	0.1820	0.0315	*V*_2_	1.3219 × 10^−4^
5	55	128.3	6	1	7	5	0.41	**0.3890**	0.0209	0.3203	0.2025	0.0672	*V*_1_	1.2689 × 10^−4^
